# Effect of Rearfoot Strikes on the Hip and Knee Rotational Kinetic Chain During the Early Phase of Cutting in Female Athletes

**DOI:** 10.1186/s40798-021-00368-w

**Published:** 2021-10-21

**Authors:** Issei Ogasawara, Yohei Shimokochi, Shoji Konda, Tatsuo Mae, Ken Nakata

**Affiliations:** 1 Department of Health and Sport Sciences, Graduate School of Medicine, Osaka University, 1-17 Machikaneyama-cho, Toyonaka, Osaka 560-0043 Japan; 2 Department of Health and Sport Management, Osaka University of Health and Sport Sciences, 1-1 Asashirodai, Kumatori-cho, Sennan-gun, Osaka, 590-0496 Japan; 3 Department of Sports Medical Biomechanics, Graduate School of Medicine, Osaka University, 2-2 Yamada-oka, Suita, Osaka 565-0871 Japan

**Keywords:** Foot strike pattern, Rotational destabilization, Horizontal-plane kinetic chain, Deceleration motion, Anterior cruciate ligament injury

## Abstract

**Background:**

Biomechanical factors affecting horizontal-plane hip and knee kinetic chain and anterior cruciate ligament (ACL) injury risk during cutting maneuvers remain unclear. This study aimed to examine whether different foot strike patterns alter horizontal-plane hip and knee kinetics and kinematics during a cutting maneuver in female athletes and clarify the individual force contribution for producing high-risk hip and knee loadings. Twenty-five healthy female athletes performed a 60° cutting task with forefoot and rearfoot first strike conditions. Horizontal-plane hip and knee moment components, angles, and angular velocities were calculated using synchronized data of the marker positions on the body landmarks and ground reaction forces (GRFs) during the task. The one-dimensional statistical parametric mapping paired t test was used to identify the significant difference in kinetic and kinematic time-series data between foot strike conditions.

**Results:**

In the rearfoot strike condition, large hip and knee internal rotation loadings were produced during the first 5% of stance due to the application of GRFs, causing a significantly larger hip internal rotation excursion than that of the forefoot strike condition. Dissimilarly, neither initial hip internal rotation displacement nor knee internal rotation GRF loadings were observed in the forefoot strike condition.

**Conclusions:**

Rearfoot strike during cutting appears to increase noncontact ACL injury risk as the GRF tends to produce combined hip and knee internal rotation moments and the high-risk lower limb configuration. Conversely, forefoot strike during cutting appears to be an ACL-protective strategy that does not tend to produce the ACL-harmful joint loadings and lower extremity configurations. Thus, improving foot strike patterns during cutting should be incorporated in ACL injury prevention programs.

**Supplementary Information:**

The online version contains supplementary material available at 10.1186/s40798-021-00368-w.

## Key Points

- Rearfoot strike induced hip internal rotation excursion at the early stance phase of cutting, not observed in the forefoot strike.
- The internally directed moment of GRF acting at the hip was the mechanical source of hip internal rotation at rearfoot contact and the hip muscle moment counteracted it.
- As a practical implication, technical optimization of the foot strike pattern during sports movement should be incorporated into the injury prevention program and risk screening testing.

## Background

Knee injuries are the second most common joint injuries after ankle injuries in team ball sports [1, 2]. Anterior cruciate ligament (ACL) injuries of any contact type (contact, noncontact, and indirect contact) account for 20.3% of all these knee injuries [3]. Female athletes have 2–4 times higher ACL injury incidence rates than male counter parts [4–7] with females of younger than 25 years old are most likely to sustain an ACL tear [8]. ACL injuries in younger individuals result in an increased risk of the early onset posttraumatic knee osteoarthritis and considerably decreased quality of life [9]. More than 70% of those occur without any direct blow to the knee from others [10] with sharp deceleration motions such as cutting and landing within short time periods from initial foot contacts in such tasks frequently associated with ACL injuries [11]. Thus, understanding the mechanisms of noncontact ACL injuries as well as risky and safe movement skills for sharp decelerating motions is essential, especially for young female athletes, to prevent sports-related ACL injuries.

Hip internal rotation combined with knee valgus and internal rotation observed at the time of noncontact ACL injuries was considered as a risky lower limb configuration for ACL injuries [10, 12, 13]. A video analysis study reported that hip internal rotation followed by subsequent knee internal rotation was commonly observed in 10 injury cases [14]. A laboratory-controlled study supported the effect of the horizontal-plane hip configuration on the knee loadings that the hip internal rotation at foot impact is a significant predictor of knee valgus loading during cutting maneuvers in female athletes [15–17]. Collectively, these previous studies suggested that the loss of rotational hip control at initial contact (IC) may indirectly alter the mechanical status of the knee, resulting in high loading on the passive knee structures including the ACL. Therefore, identifying the mechanical and technical factor that induces the horizontal hip and knee rotational instability is very interesting from the perspective of ACL injury risk.

Given the noncontact mechanism of ACL injuries, the ground reaction force (GRF) exerted at the center of pressure (CoP) of the landing foot would be a primary external force which is capable of developing the abnormal hip and knee rotational configurations in this time frame. Previous laboratory-controlled studies have tried to identify the specific joint loading patterns associated with the risky hip and knee configurations [16–19]. In the majority of these studies, only the resultant moment acting on the joint of interest was evaluated as a measure of joint loading; however, how each of the external force component, such as translational and rotational inertia, gravity, free moment and GRF, developed that resultant moment was not well focused on in the context of ACL injury. Specifically, knowing the GRF’s contribution on the final joint kinematics may provide an insight into the mechanical cause of the hip and knee kinetic chain observed in the situation of the noncontact ACL injury.

The rearfoot strike (RFS), as a potential technical factor, has been reported to be frequently associated with noncontact ACL injuries [14, 20–22]. Laboratory-controlled studies have also suggested that the different foot strike pattern [23, 24] or foot orientation relative to the floor [25, 26] altered the knee loading pattern during cutting or landing maneuvers. Further, our group recently reported that the GRF acting at the rearfoot is more likely to apply to the combined knee valgus and tibial internal rotation moment in the early phase of cutting maneuvers [27]. Although the previous studies revealed the link between the foot strike pattern and knee loading pattern, it is still unknown whether different foot strike patterns affect the hip and knee horizontal-plane kinetics and kinematics during the deceleration phase of cutting maneuvers in female athletes.

Therefore, the purpose of this study was to investigate whether the different foot strike patterns, forefoot strike (FFS) and RFS, alter the horizontal-plane hip and knee kinetics and kinematics during a cutting maneuver in female athletes. Based on the potential risks associated with RFSs, we hypothesized that the RFS would produce a significantly greater GRF-driven internal rotation moment of hip and knee than FFS, and this GRF loading would produce greater hip and knee internal rotation angular excursions and angular velocities than the FFS during the early phase of the cutting maneuver.

## Methods

### Ethics Statement

The ethics board approved this experiment (Mukogawa Women’s University, No. 12–13), and written informed consent was obtained from all participants. This research was performed in accordance with all relevant guidelines and regulations and in accordance with the Declaration of Helsinki.

### Participants

Twenty-five healthy female collegiate handball players participated in this study. Athletes’ mean height was 160.5 (standard deviation [SD] 5.1) cm, mean mass was 55.6 (SD 5.6) kg, and mean age was 21.2 (SD 0.9) years. All athletes belonged to the university’s handball team, which usually participates in the Japanese collegiate top tournament yearly. Participants had more than five years of handball experience, trained 2 − 3 h per day, and were familiar with the change of direction movements. All the participants’ normal foot strike pattern for the cutting limb was the forefoot strike. Athletes who had a history of severe knee injuries, such as an ACL tear, or minor lower limb trauma, such as an ankle sprain, in the 6 months preceding the date of initiation of the experiment were excluded.

### Procedure

**Fig. 1:**
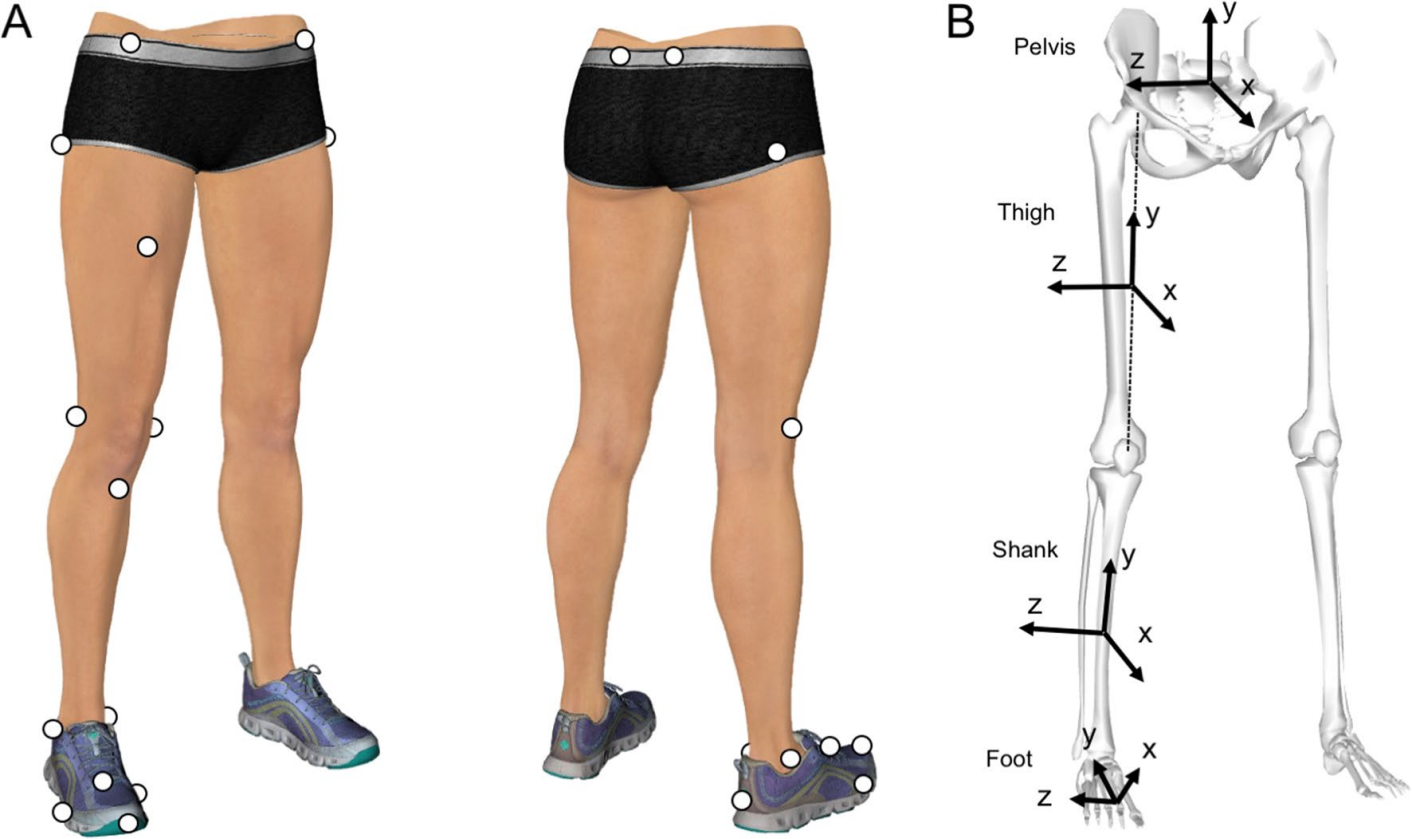
Reflective marker attachment site and orientations of the local coordinate systems. **A** Location of reflective markers on the right leg. **B** The kinematic model consists of 4 segments (foot, shank, thigh, and pelvis) and 3 joints (ankle, knee, and hip). The local coordinate system for each segment is defined as a 3 × 3 rotation matrix consisting of 3 common perpendicular unit base vectors: the x-axis is the anterior/posterior axis pointing forward, y-axis is the vertical axis along with the longitudinal line of the segment pointing upward, and z-axis is the medial/lateral axis pointing to the right of the segment

**Fig. 2:**
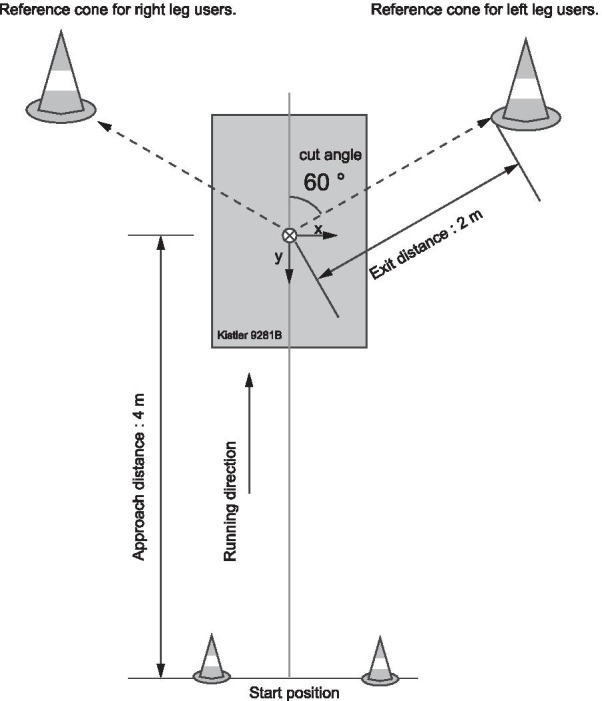
Top view of experimental platform. The participants were instructed to run on the wooden platform and change the running direction with a single step (cutting task) at an angle of 60 degrees. The approach distance from the start position to the center of the force plate was 4 m. The reference cone was placed on the floor at 2 m from the approach line at an angle of 60 degrees to guide the participant. The exit distance (from the center of the force plate and the reference cone) was 2 m

Seventeen reflective markers (diameter, 14 mm) were attached to 17 landmarks (Fig. 1A, Table 1) by the same researcher (IO). The participants were instructed to run on the wooden platform and change the running direction with a single step (cutting task) at an angle of 60° [27, 28]. The approach distance from the start position to the center of the force plane was 4 m. The reference cone was placed on the floor 2 m from the approach line, at an angle of 60° to help guide the participant in this movement (Fig. 2). The test leg was determined by asking the participants which leg was preferred to control the foot-floor contact point at the instance of impact, and a total of 23 participants chose their right leg, while 2 selected their left leg. Athletes used their own ASICS handball shoes to facilitate precise foot control and their safety; therefore, the shoe–surface friction coefficient was not strictly controlled among the athletes.

**Table 1:** Locations of the reflective body markers on the cutting limb and pelvis

Toe	On the most anterior point of the toe, over the shoe
Medial toe	Medial aspect of the most prominent point of the first MP joint, over the shoe
Lateral toe	Lateral aspect of the most prominent point of the fifth MP joint, over the shoe
Dorsal foot	Dorsal aspect of the midpoint of the second and third MP joints, over the shoe
Medial ankle	On the most prominent point of the medial malleolus
Lateral ankle	On the most prominent point of the lateral malleolus
Heel	On the most posterior point of the shoe heel and 2 cm above the floor level when the subject is standing stationary
Medial knee	On the most prominent point of the medial femoral epicondyle
Lateral knee	On the most prominent point of the lateral femoral epicondyle
Tibial tuberosity	On the most prominent point and anterior aspect of the tibial tuberosity
Great trochanter	On the most prominent point of the great trochanters (both sides)
Mid-thigh	On the halfway (approximately) point of the anterior aspect of the thigh
ASIS	On the most prominent point of the ASISs (both sides)
PSIS	On the most prominent point of the PSISs (both sides)

ASIS, anterior superior iliac spine; PSIS, posterior superior iliac spine; MP, metatarsophalangeal

Two different foot strike conditions (RFS versus [vs.] FFS) were tested. In the RFS condition, participants were required to hit the force plate with their heel first to locate the CoP of the rearfoot at the beginning of the stance phase and then move their weight to the forefoot to push off. For the FFS condition, participants were asked to touch the force plate with their forefoot throughout the stance phase. Since we acknowledged that the GRF acting at the rearfoot more frequently applies the combined knee valgus and tibial internal rotation loads, the approach speed was controlled at less than 2.0 m/s and the athlete was asked to perform the task with 60–70% of maximum intensity for their safety (Refer to Ogasawara et al. [27] for the theoretical explanation for approach speed determination). To specify the characteristics of cutting motion, we requested the athletes to resemble the faking action, which is typically used during one-on-one handball games. No further specific instruction was provided to measure athletes’ natural cutting performance.

After task familiarization was complete, a static standing pose was recorded as the reference for neutral joint angles, and 10 successful trials for each foot condition were recorded. To confirm whether the approach speed was less than 2.0 m/s for each trial, the approach speed was monitored online with the data streaming feature of the motion capture system (NatNet, Motive Body 1.1, NaturalPoint, Inc., Corvallis, OR, USA) using a custom-made LabVIEW script (National Instruments, Austin, TX, USA). The approach speed was defined as the average speed of the midpoint of both ASIS markers from -40 to 0 ms before the initial foot contact. A 3-min rest between conditions and an approximately 30-s interval between trials were provided to minimize fatigue. To equalize the possible effect of fatigue between foot strike conditions, the order of the 2 conditions was randomized for each participant. The success of the foot strike pattern was visually judged by 2 researchers (IO and assistant YK, CA or KM) for each trial. If a consensus was not achieved, that trial was discarded, and the participant was requested to execute an additional trial. The three-dimensional marker positions were captured using 12 optical cameras (OptiTrack S250e with 250-Hz sampling; software: Motive Body 1.1, NaturalPoint, Inc.) simultaneously with the GRF recordings (force plate: type 9281B, Kistler, Winterthur, Switzerland; data acquisition device: USB-6218 BNC with 1-kHz sampling, National Instruments, Austin, TX, USA). A clock device (eSync, NaturalPoint, Inc.) was used to generate the timing signal to synchronize the onset of the camera and force plate recordings.

### Data Processing

**Fig. 3:**
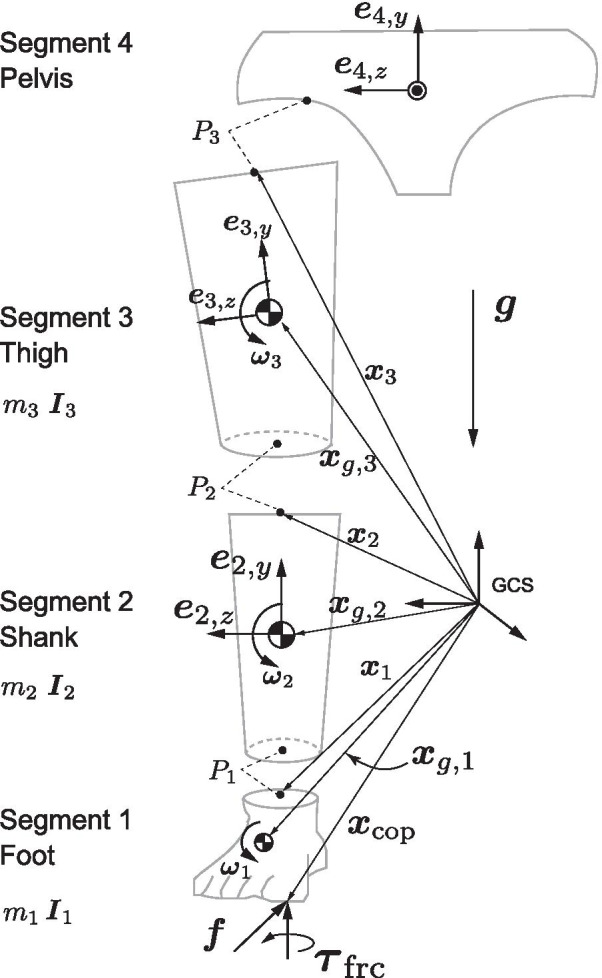
Three-dimensional four-link kinematic model. Schematic frontal view of the kinematic model used in this study. For visibility, each segment is visualized separately at the joint, but they are actually connected

The position data were smoothed using a second-order low-pass zero-lag digital Butterworth filter at cutoff frequencies of 12–15 Hz, which were determined by residual analysis [29]. The GRF data were smoothed at the same cutoff frequencies as those used for smoothing the marker data [30]. The stance phases (vertical GRF > 10 N) of the position and GRF data were extracted and time-normalized (0–100%) throughout the stance phase. The kinematic model was consisted of 4 segments (foot, shank, thigh, and pelvis) with 3 joints (ankle, knee, and hip) (Fig. 3). The ankle and knee joint centers were calculated as the midpoint between medial and lateral markers for the ankle and knee joints. The hip joint center was calculated using a previously reported method [31]. The local coordinate system (LCS) of each segment was defined as a 3 × 3 rotation matrix consisting of common perpendicular unit base vectors where the x-axis (${{\varvec{e}}}_{i,x}$) was the anterior/posterior axis pointing to the anterior of the segment, y-axis (${{\varvec{e}}}_{i,y}$) was the vertical axis along with the longitudinal line of the segment pointing upward, and z-axis (${{\varvec{e}}}_{i,z}$) was the medial/lateral axis pointing right of the body [32, 33]. The segmental mass, position of mass center, and inertia moment were estimated based on the data of Japanese athletes [34]. The hip and knee internal(+)/external(−) rotations were defined as the segmental rotation around the y-axis of the thigh and shank segments, respectively, and those angles were calculated using Derrick et al.’s [35] method for the hip joint and Grood and Suntay’s method [32] for the knee joint. The calculated internal/external rotation angles were offset with the angle at the static pose. In addition, the internal/external rotation angular excursions were calculated by subtracting with the angle at IC of each trial to quantify the angular displacements that occurred after foot contact. The internal/external rotational joint angular velocities of the hip and knee were obtained by using the numerical differentiation of the joint angle. See Additional file 1 for the detail of joint angle and angular velocity calculation.

The Newton–Euler equation of motion was solved to obtain all of the moment components, e.g., (1) resultant moment, (2) moment of GRF, (3) rotational inertia moment, (4) gyroscopic moment, (5) moment of linear inertial force, (6) moment of gravity acting at the segmental mass center, and (7) joint moment due to a friction moment acting around the vertical axis at CoP (See Additional file 1: Figure S1 for the free-body diagram and the mathematical detail of the inverse dynamics model).

For each moment component, the projections onto the y-axis of the thigh and shank segments were calculated to extract the internal(+)/external(−) rotation components. This process corresponded to express the resultant moment in the LCS of the distal segment [35]. According to the joint structure, the resultant hip internal/external rotation moment was assumed to be produced mainly by the hip rotators to balance the external loadings. In contrast, the resultant knee internal/external rotation moment was regarded as a resistive moment derived from the stretched passive structures including the ACL [35], under the presence of the external loadings on the knee. All the numerical calculations were performed with Scilab 6.0.0 custom scripts (https://www.scilab.org/).

### Statistical Analysis

An a priori power analysis using G*Power 3.1.9.4 suggested that the appropriate sample size for the paired t test was n = 22 to achieve 80% power at a statistical significance criterion of 0.01, with a large effect size (ES) (Cohen *d* > 0.8). The degree of ES was expected from the previous literature of our laboratory [27].

To confirm whether the approach speeds differed between foot strike conditions, the average speeds of the midpoint between both anterior superior iliac spine markers from − 40 to 0 ms before foot contact were compared using the paired t test (*P* < 0.05).

To examine the contribution of each moment component on the magnitude of the resultant hip and knee moments, the time-series data of each moment component were normalized by the resultant moment in a point-by-point manner and averaged over the stance phase (0–100%) as

$$c_{j} = \frac{1}{101}\mathop \sum \limits_{i = 0}^{100} \left( {\tau_{j,i} /\tau_{RES,i} } \right),$$

where $c_{j}$ is the relative contribution, $\tau_{j}$ is the each moment component (j = 1:resultant, j = 2:GRF, j = 3:rotational inertia, j = 4:gyroscopic, j = 5:linear inertia, j = 6:gravity, and j = 7:friction moment), and $\tau_{RES}$ is the resultant moment. Two-way analysis of variance (ANOVA) (moment components × foot strike conditions) with a post-hoc Tukey’s honestly significant difference test was conducted to determine the component-wise difference of contribution $c_{j}$ (*P* < 0.01).

Time-series changes in joint angles, angular excursions, angular velocities, and moment component were compared between foot strike conditions using the statistical parametric mapping (SPM) two-tailed paired t test [36]. The alpha level for the SPM test was set at 0.01. To quantify the ES of significant difference, the point-by-point Cohen d value was calculated and averaged over the phase when the SPM test detected a significant difference. Based on previously published data from our laboratory which compared the knee internal/external rotation moment between FFS and RFS, significant differences with a Cohen d value of > 0.8 were regarded as a large ES [27]. All SPM tests were implemented using the spm1d code (www.spm1d.org) in Python 3.6.3 (https://www.python.org/downloads/release/python-363/).

## Results

### Approach Speed

The average approach speed was not significantly different between the foot contact conditions (RFS: 1.47 [SD 0.29] m/s, FFS: 1.50 [SD 0.34] m/s, *P* = 0.21, Cohen *d* = 0.09). All the trials satisfied with the safety criteria of approach speed < 2.0 m/s.

### Joint Moment

**Fig. 4:**
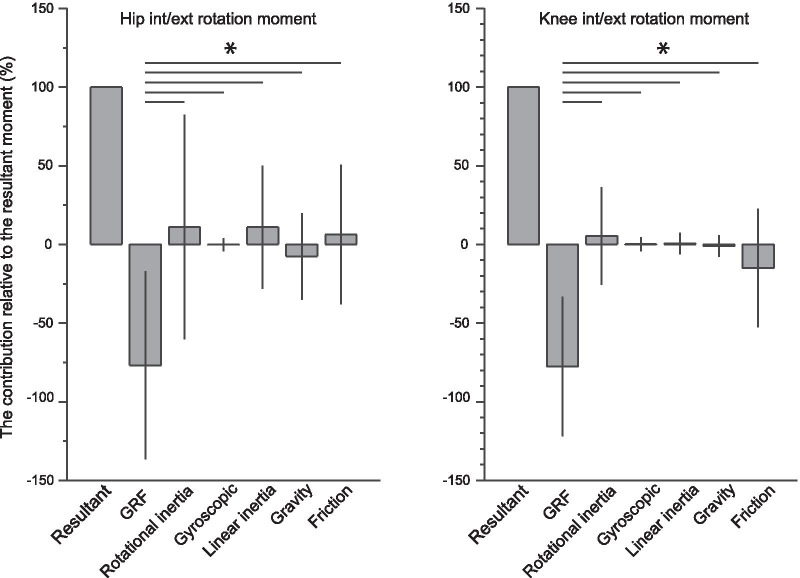
Contribution of the moment variables relative to the magnitude of the resultant moment. Since the two-way analysis of variance suggested that the foot strike condition has no significant effect, a post-hoc Tukey’s honestly significant difference test is used without consideration of the foot strike condition and displayed here. The results indicate that the moment of GRF has a dominant but negative contribution to the resultant moment, whereas the other moment variables show a small contribution to rotational kinetics of the hip and knee. Asterisk denotes a significant difference. GRF: ground reaction force

Two-way ANOVA demonstrated a significant main effect of moment components (hip: *P* < 0.01, F = 650.0; knee: *P* < 0.01, F = 1936.9) on the contribution to the magnitude of the resultant hip and knee moments but not the foot strike conditions (hip: *P* = 0.75, F = 0.095; knee: *P* = 0.07, F = 3.22). A significant interaction between the foot strike conditions and moment components was found in the knee (*P* < 0.01, F = 3.59) but not in the hip (*P* = 0.15, F = 1.59). For the hip and knee, the moments of GRF showed large but opposite contributions relative to the resultant moments, indicating that those 2 moment components were almost counterbalanced (Fig. 4). Additionally, since the contributions of the other moment components were significantly small relative to the moment of GRFs, this study focused on the resultant moments and moments of GRF for the report of the SPM test.

**Fig. 5:**
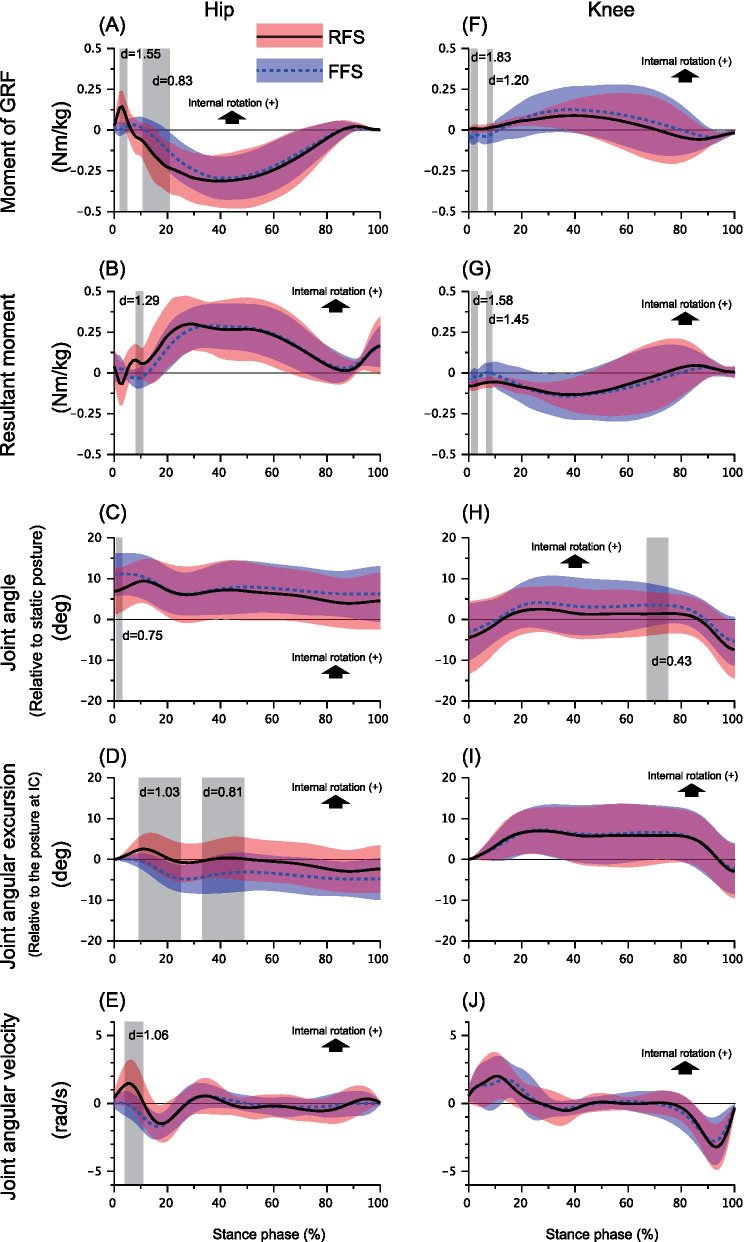
Comparison of kinetic and kinematic variables between foot strike patterns with SPM. Ensemble averages (standard deviations) of the moment of GRF (**A**, **F**), resultant moment (**B**, **G**), joint angle (**C**, **H**), angular displacement (**D**, **I**), and angular velocity (**E**, **J**) of the hip (left column) and knee (right column). On each panel, the solid line with orange shadow indicates the rearfoot strike condition, whereas the dashed line with the blue shadow illustrates the forefoot strike condition. The gray shaded durations are the phases where the statistical parametric mapping two-tailed paired t test detected a significant difference with an alpha level less than 0.01. For each significant difference, the Cohen d value is calculated as an effect size. The critical thresholds are shown on each panel. (For interpretation of the references to color in this figure legend, the reader is referred to the web version of this article). GRF: ground reaction force

For the hip and knee, the moments of GRF and resultant moments showed similar temporal patterns but in the opposite direction (Figs. 4B, F, G, 5A). For the hip joint, the RFS produced a rapid increase of internally directed moment of GRF during the first 5% of the stance phase and suddenly switched to externally directed moment during the next 10–20% of the stance phase (Fig. 5A). The internally directed moment of GRF at hip was significantly greater with RFS than with FFS, and it showed a very large ES (RFS: 0.12 [SD 0.03] vs. FFS: 0.01 [SD 0.00] Nm/kg, Cohen *d* = 1.55, *P* = 0.032, Fig. 5A). The externally directed hip resultant moment in RFS counteracted the initial internally directed moment of GRF during the first 5% of the stance phase; however, there was no significant difference between foot strike conditions during this phase (Fig. 5B). At approximately 10% of the stance phase, RFS showed significantly greater internally directed hip resultant moment than FFS did (RFS: 0.07 [SD 0.00] Nm/kg vs. FFS: − 0.03 [SD 0.00] Nm/kg, Cohen *d* = 1.29, *P* = 0.014, Fig. 5B).

Moment of GRF at the knee joint was in the opposite direction with RFS and FFS during the first 10% of the stance phase (e.g., RFS was internal, whereas FFS was external), and the ES was very large (RFS: 0.01 [SD 0.00] Nm/kg vs. FFS: − 0.04 [SD 0.00] Nm/kg, Cohen *d* = 1.83, *P* = 0.043, and RFS: 0.02 [SD 0.00] Nm/kg vs FFS: − 0.03 [SD 0.01] Nm/kg, Cohen *d* = 1.20, *P* = 0.03, Fig. 5F). The directions of the resultant moment in both foot strike conditions were externally directed, and the RFS showed significantly greater magnitude at about 1–10% of the stance phase with large ESs (RFS: − 0.07 [SD 0.00] Nm/kg vs. FFS: − 0.02 [SD 0.00] Nm/kg, Cohen *d* = 1.58, *P* = 0.049, and RFS: − 0.06 [SD 0.00] Nm/kg vs. FFS:0.002 [SD 0.00] Nm/kg, Cohen *d* = 1.45, *P* = 0.027, Fig. 5G].

### Joint Angles and Joint Angle Excursions

The hip was in an internally rotated position throughout the stance phase for both foot strike conditions, and there was no large ES significant difference in the hip joint angle between foot strike conditions (Fig. 5C). However, the RFS caused a greater hip internal rotation excursion after IC, showing a significant difference with a large ES during the 5–49% of the stance phase as compared to the FFS (RFS: 1.5 [SD1.0] deg vs. FFS: − 2.14 [SD 1.20] deg, Cohen *d* = 1.03, *P* < 0.001, and RFS: 0.07 [SD 0.33] deg vs. FFS: − 3.71 [SD 0.49] deg, Cohen *d* = 0.81, *P* < 0.001, Fig. 5D). The knee showed similar rotational patterns in both foot strike conditions. There was no large ES significant difference in the knee rotational angle and excursion between the foot strike conditions (Fig. 5H, 4I).

### Joint Angular Velocity

The RFS showed an abrupt increase of internally directed hip angular velocity after IC, which was significantly higher than that of FFS during the 4–11% of the stance phase (RFS: 1.21 [SD 0.23] rad/s vs. FFS: − 0.18 [SD 0.24] rad/s, Cohen *d* = 1.06, *P* < 0.001, Fig. 5E). The knee angular velocities in both foot strike conditions were commonly internally directed during the first 30% of the stance phase and externally directed during the last 20% of the stance phase, with no significant difference between the conditions (Fig. 5J).

## Discussion

This study is the first to report that the foot strike pattern differentiated the horizontal-plane hip kinetics and kinematics as well as knee kinetics during the early stance phase of a cutting maneuver in female athletes. Specifically, the RFS showed a rapid increase of internally directed moment of GRF at the hip during the first 5% of the stance phase, followed by a subsequent increase of the hip internally directed joint angular velocity during 4–11% of the stance phase, and hip internal rotational excursion peaking at approximately 10% of the stance phase (Fig. 5A, E, D). Those characteristics were not found in the FFS condition. The critical difference found in the knee kinetics was that the RFS and FFS demonstrated the opposing directions of the moment of GRF during 1–10% of the stance phase, i.e., the RFS produced the internally directed, while the FFS showed the externally directed moment of GRF (Fig. 5F, G). For both hip and knee joint, the moments of GRF were the significant determinants of the resultant joint moments (Fig. 4), providing insight into the generation of the high-risk hip and knee loading patterns during cutting in females. Although our results did not show the significant difference in the knee kinematic variables between foot strike patterns, the findings generally supported our hypotheses.

The key finding in the kinetic outcomes was that the hip and knee loading patterns (direction of the moment of GRF at hip and knee) was different between foot strike patterns and this difference may explain the discrepancy in the ACL injury risk associated with the foot strike pattern. The RFS exhibited a combination of internally directed hip and knee moments of GRF at the first 10% of stance (Fig. 5A, F). Conversely, the FFS showed the combination of externally directed hip and knee moments of GRF at the same time frame (Fig. 5A, F). The increased hip internal rotation excursion in female athletes during the functional movements has been interpreted as a risk factor for ACL injury [15–17] because the hip internal rotation in weight-bearing situation results in the subsequent knee valgus kinetic chain [17]. In addition, the internal tibial rotation moment has been reported to increase the in-situ force on the ACL [37] and is associated with the mechanism of ACL injury [38, 39], whereas the tibial external rotation has opposite mechanical effect on ACL [40]. In this study, since we used a slower approach speed (< 2.0 m/s) than previous cutting studies [15, 41], although significant differences of hip and knee rotational GRF loading pattern with the opposite directions were found between the foot strike patterns, the mechanical load may not be sufficient to cause the actual knee kinematic difference between foot strike conditions. Hence, the findings of this study alone cannot conclude that the different foot strike strategy principally determines the final knee kinematics observed in ACL injuries [38]. However, at least in the kinetic level, the hip and knee joints were sensitive to foot strike pattern difference even at a slower approach speed. Therefore, from the perspective of the horizontal-plane hip and knee kinetic chain, it is suggested that the RFS potentially adds to the risk of ACL injury, whereas the FFS is relatively an ACL-protective foot landing technique.

In this study, the hip internal rotation excursion in RFS showed a temporal increase peaking around 10% of stance but it did not maintain over the stance phase (Fig. 5D). Similarly, the temporal hip internal rotation at early stance phase was consistent among the laboratory-controlled cutting studies which the ACL injury was not observed during their experiments [42, 43]. This implied that, in the previous laboratory-controlled studies as well as this study, the magnitude of hip internal rotation moment due to GRF was not large enough to develop a complete hip internal displacement which was sufficient to threaten the ACL. The quantitative video analyses of the actual ACL injury event have demonstrated that the hip internal rotation continued until the ACL injury occurred [14]. In contrast, the video analysis of the high-risk motion with non-injury situation demonstrated no prominent hip internal rotations within 40 ms after IC as well as the knee valgus and tibial internal rotation [44]. Therefore, it may be speculated that the presence of the complete hip internal rotation at the beginning of the stance phase determines the subsequent stance limb configuration and affects the risk of ACL injury.

Limited passive range of motion (RoM) for hip internal rotation has been reported to be associated with the incidence of noncontact ACL injury [45] and rerupture of the ACL [46]. This information seems to be contradictory to our aforementioned proposal. However, we believe that those clinical findings and our proposal do not conflict because the time scopes were different. As aforementioned, we suggested that the initial hip internal rotation due to internally directed GRF moment acting at the hip may be problematic for subsequent alignment control; therefore, this concern would be pronounced in the pre-injury phase (e.g., period from IC to ligamentous rupture). Contrary to this, a narrow hip rotational passive RoM may become problematic at about the terminal of hip passive RoM since cadaveric studies demonstrated that the knee rotational stress increases as the hip rotation is mechanically constrained by the limit of passive RoM [47]. Hence, these mechanics may occur around the “rupture phase” of ACL injury. It could also be speculated that if the narrow hip internal rotation passive RoM reflects the dysfunction of hip external rotator muscles, such as contracture by muscle fatigue, it may not adequately counteract the internally directed GRF loading acting at the hip at the foot impact phase. However, the effect of passive structures, such as an increased femoral alpha angle in the anteroposterior view [48], could also contribute to the limited hip internal passive RoM and be associated with noncontact ACL injury; therefore, the dysfunction of hip external rotators alone does not fully explain the association between the static measure of hip rotation passive RoM and the mechanism of ACL injury. Further study about the relationship between the hip passive RoM and dynamic hip and knee kinetic chain during functional activities will be needed to clarify this issue.

The component-wise analysis of the equation of motion demonstrated that the externally directed hip resultant moment (muscle moment) counteracted the internally directed moment of GRF exerted at the hip (Fig. 5A, B), suggesting that adequate hip muscular control was essential to maintain a neutral hip orientation after IC of the cutting maneuver. This was also supported by our results that the magnitudes of the other moment components were too small to balance the moment of GRF, suggesting that the hip muscle moment was a primary counterpart of the moment of GRF (Fig. 4). If the hip rotator muscles failed to increase the horizontal-plane joint impedance prior to foot impact and could not adequately balance the internally directed moment of GRF exerted at the hip, the initial hip internal excursion would become more pronounced. Leetun et al. reported that female athletes had significantly decreased hip external rotation isometric strength (percentage body weight) than male athletes, and they prospectively revealed that hip external rotation strength was the effective predictor of the likelihood of sustaining noncontact injuries, including ACL rupture [49]. Omi et al. suggested the use of a “hip-focused” training to increase hip rotational strength and hip neuromuscular control; they achieved 62% reduction of noncontact ACL injuries in female basketball players [50]. Collectively, these clinical findings highlight the importance of hip external rotator strength for managing the risk of ACL injury [17]. Our results support the mechanical rationale underlying those previous findings regarding how hip external rotator moments contribute to the control of hip rotational kinematics during athletic maneuvers.

Regarding practical applicability, our findings may be utilized in injury risk profiling and technical intervention with an aim to reduce the relative risk of ACL injury. Identifying habitual forefoot/rearfoot users during horizontal deceleration can be a field risk screening method. This possibility is further supported by video studies, which reported that the rearfoot strike was the common inciting motion in noncontact ACL injuries [14, 20, 22, 51]; additionally, a prospective regional analysis of in-shoe forces using a foot pressure sensor revealed that an athlete who would go on to rupture his ACL had a rearfoot strike tendency in running and cutting maneuvers [52]. The advantages of the forefoot strike can be used as a practical indicator of foot strike technique modification in an ACL injury mitigation training program. Our group previously reported that the forefoot strike in cutting dramatically reduced the probability of producing the combined knee valgus and tibial internal rotation loading compared to the rearfoot strike [27]. To facilitate the use of forefoot landing in normal performance, not only localized foot orientation control but also whole-body spatiotemporal motor controls—such as successful velocity control during the penultimate foot contact prior to the final plant foot contact [53], the stable control of the trunk and shank orientation relative to the floor [54], and appropriate stance width not increasing the offset distance from the body center of mass (CoM) to the margin of base of support (BoS) [54]—may be important. The ACL injury prevention program should incorporate these technical aspects to reduce the less preferable hip and knee kinetic pattern associated with RFS during cutting motions.

There were several limitations to this study. First, the current results were from female participants, so caution is needed when generalizing our findings to male athletes. Sex disparities are known to exist in the hip and knee biomechanics during cutting maneuvers [16]; thus, this study used female participants to isolate the effect of foot strike pattern. Second, despite being warranted by the participants’ safety, the slow approach speed used in our study was not reflective of a practical sports situation, and it must be noted that the magnitudes of the hip and knee kinetic and kinematic outcomes sampled from our athletes may underestimate those in the real-world cutting motions. Specifically, the mechanical load in our experiment might not be sufficient to evaluate the effect of different foot strike patterns on the internal/external knee rotation kinematics since there was no significant difference between foot strike conditions. However, from the kinetic perspective, the temporal patterns of the hip and knee kinetics are consistent with the results of previous similar cutting studies [55], suggesting the results of the GRF loading patterns (direction of the GRF moments on the hip and knee) were not affected by our slow approach speed. Third, since our cutting experiment used the planned cutting task, this study cannot address the effects of an athlete’s instantaneous decision making and/or attention sharing in switching the foot strike pattern. Previous studies that examined the effect of decision making on the choice of cutting direction reported that the magnitude of knee joint moment (loading) was significantly greater in the unanticipated cutting condition than that of the planned condition, suggesting that cognitive demand may affect the biomechanical status during athletic tasks [56]. Although the aim of this study was not to examine the effect of decision making on selecting the appropriate foot strike strategy in the cutting task, to allow the practical implementation of our findings, studying an unanticipated foot strike switching strategy is worth investigating to reproduce an athlete’s demands in a practical sports environment. Future studies should focus on this difference between planned and unplanned cutting task.

## Conclusions

In conclusion, the RFS showed a combination of greater hip internal rotation angular velocity and hip internal joint excursion combined with the increased internally directed moment of GRF at the hip and knee during the early phase of the cutting task, which was not seen in FFS. Such a hip and knee kinetic combination in the RFS may potentially explain the known discrepancy in ACL injury between the foot strike patterns. As a practical implication of this study, the prevention strategy should incorporate any possible technical interventions to achieve an appropriate foot strike strategy, for example, the penultimate foot contact before final foot placement, the stable control of trunk orientation, and making a narrow stance width to prevent increasing the CoM to BoS offset. The intervention should also focus on the external hip rotator muscle function to counteract the GRF-induced initial hip internal excursion, if the athlete encounters the unavoidable RFS in sports.

## Supplementary Information

**Additional file 1**. Figure S1 for the free-body diagram and the mathematical detail of the inverse dynamics model.

## Data Availability

Data that support the findings of this study will be made available from the corresponding author upon reasonable request.

## Abbreviations

ACL: Anterior cruciate ligament
GRF: Ground reaction force
CoP: Center of pressure
FFS: Forefoot strike
RFS: Rearfoot strike
ASIS: Anterior superior iliac spine
PSIS: Posterior superior iliac spine
MP: Metatarsophalangeal
LCS: Local coordinate system
ES: Effect size
SPM: Statistical parametric mapping
